# Biomass for energy in the European Union - a review of bioenergy resource assessments

**DOI:** 10.1186/1754-6834-5-25

**Published:** 2012-04-30

**Authors:** Niclas Scott Bentsen, Claus Felby

**Affiliations:** 1 Faculty of Life Sciences, University of Copenhagen, Rolighedsvej 23, Copenhagen, DK-1958, Frederiksberg, Denmark

**Keywords:** Bioenergy, Biomass resource potential, Land use

## Abstract

This paper reviews recent literature on bioenergy potentials in conjunction with available biomass conversion technologies. The geographical scope is the European Union, which has set a course for long term development of its energy supply from the current dependence on fossil resources to a dominance of renewable resources. A cornerstone in European energy policies and strategies is biomass and bioenergy. The annual demand for biomass for energy is estimated to increase from the current level of 5.7 EJ to 10.0 EJ in 2020. Assessments of bioenergy potentials vary substantially due to methodological inconsistency and assumptions applied by individual authors. Forest biomass, agricultural residues and energy crops constitute the three major sources of biomass for energy, with the latter probably developing into the most important source over the 21^st^ century. Land use and the changes thereof is a key issue in sustainable bioenergy production as land availability is an ultimately limiting factor.

## Introduction

**Figure 1:**
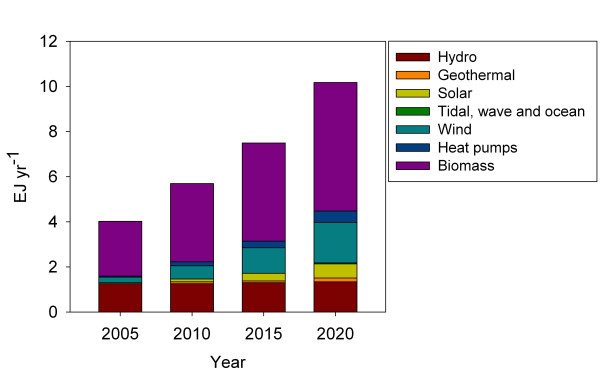
Projections on the stipulated production of energy from renewable resources in the EU27 countries based on national renewable energy action plans [76].

Energy security and climate change mitigation are core elements in current European energy policy. The EU countries are mandated to meet by 2020 a target of 20% renewable resources in the energy supply and 10% renewable resources in energy in the transport sector [1]. The latter corresponds to a replacement of 50 billion litres of fossil transportation fuels. The Energy Strategy 2020 [2] of the European Commission calls for increased use of renewable resources in the energy system and the European Council has presented a long term target for the EU and other industrialised countries of 80 to 95% cuts in greenhouse gas emissions by 2050. A cornerstone in renewable energy projections of the European Union is biomass, which is expected to account for 56% of the renewable energy supply in the EU27^a^ by 2020 (Figure 1).

**Table 1:** Characteristics of studies included in the summary of European bioenergy potentials

**Reference**	**Year of publication**	**Type of publication†**	**Biomass resources ‡**	**Type of potential #**	**Temporal scope**	**Geographical scope**
Asikainen [59]	2008	Report	FO	Theoretical + technical	2000-10	EU27
Böttcher [39]	2010	Report	AR, FO	Theoretical + technical	2010	EU27
Ericsson [41]	2006	Journal	EC, AR, FO	Technical	2015-25, 2025–45, 2045-	EU25
Fischer [16]	2001	Journal	EC, AR	Technical-sustainable	1990, 2050	EU27 + (CH, NO, IS and Balkan), -(Baltic states)
Fischer [44]	2010	Journal	AR	Technical-sustainable	2000, 2030	EU27
Haberl [18]	2010	Journal	EC, AR, FO	Technical-sustainable	2050	Geographical Europe –(former Soviet states)
Hoogwijk [23]	2005	Journal	EC	Technical	2050, 2100	EU27 + (CH, NO, IS and Balkan), -(Baltic states)
Mantau [60]		Report	FO	Theoretical + technical	2010, 2020, 2030	EU27
Panoustou [56]	2009	Journal	AR, FO	Technical	2000, 2010, 2020	EU27
RENEW [53]	2008	Report	EC, AR, FO	Technical	2000-09, 2020	EU27 + CH, -(CY, MT)
Scarlat [49]	2010	Journal	AR	Technical-sustainable		EU25
Siemons [50]	2004	Report	EC, AR, FO	Technical-economic	2000, 2010, 2020	EU27
Smeets [30]	2007	Journal	EC, AR	Technical	2050	EU27 + (CH, NO, IS and Balkan), -(Baltic states)
Panoustou [56]	2009	Journal	AR, FO	Technical-economic	2000, 2010, 2020	EU27
Smeets [31]	2007	Journal	FO	Technical	2050	EU27 + (CH, NO, IS and Balkan),
Van Vuren [35]	2009	Journal	FO	Technical-economic	2050	EU27 + (CH, NO, IS and Balkan),

† ‘Report’: Data published in a report. ‘Journal’: Data published in refereed journals.

‡ ‘EC’ = dedicated energy crops,’AR’ = agricultural residues, ‘FO’ = forest biomass.

# The distinction between different types of biomass resource potentials is explained below; ‘+’ = two different resource potentials are estimated, ‘-‘= type of resource potential cannot be assigned one category.

Government programmes towards increased use of renewable resources for energy are not exclusive to Europe. In the United States the Energy Policy Act [3] and the Energy Independence and Security Act [4] focus on promoting various renewable resources, wind, solar, hydro, geothermal and within biomass mainly liquid biofuels and sets a target of 136 billion litres of biofuel for transport in 2022, hereof 80 billion litres from advanced biofuels, not based on corn starch. The Brazilian ProAlcool programme from 1975 [5] has contributed to an increased Brazilian ethanol production from below 1 billion litres in 1975 to 26 billion litres in 2009/10 [6-8].

The global perspectives for future energy production are on the use of more renewable resources in general and on biomass in particular [9]. In the European Union the overall target for renewable energy is higher than anywhere else.

### Biomass supply for energy in the EU

Numerous studies on biomass/bioenergy resources on global [10-36] and European [16,18,23,30,37-56] level have been published over the last 20 years. This review concerns European bioenergy resources only. For comprehensive reviews on global bioenergy levels Berndes et al. [57] and Offermann et al. [58] may be consulted. In the following we summarize estimates of biomass for bioenergy resources on a European level (Table 1). The geographical coverage is the EU25,^b^ EU27 or geographical Europe excluding former Soviet states. The impact of various geographical scopes is discussed in the subsequent section. All resource potentials are expressed as lower heating value of the primary biomass.

**Figure 2:**
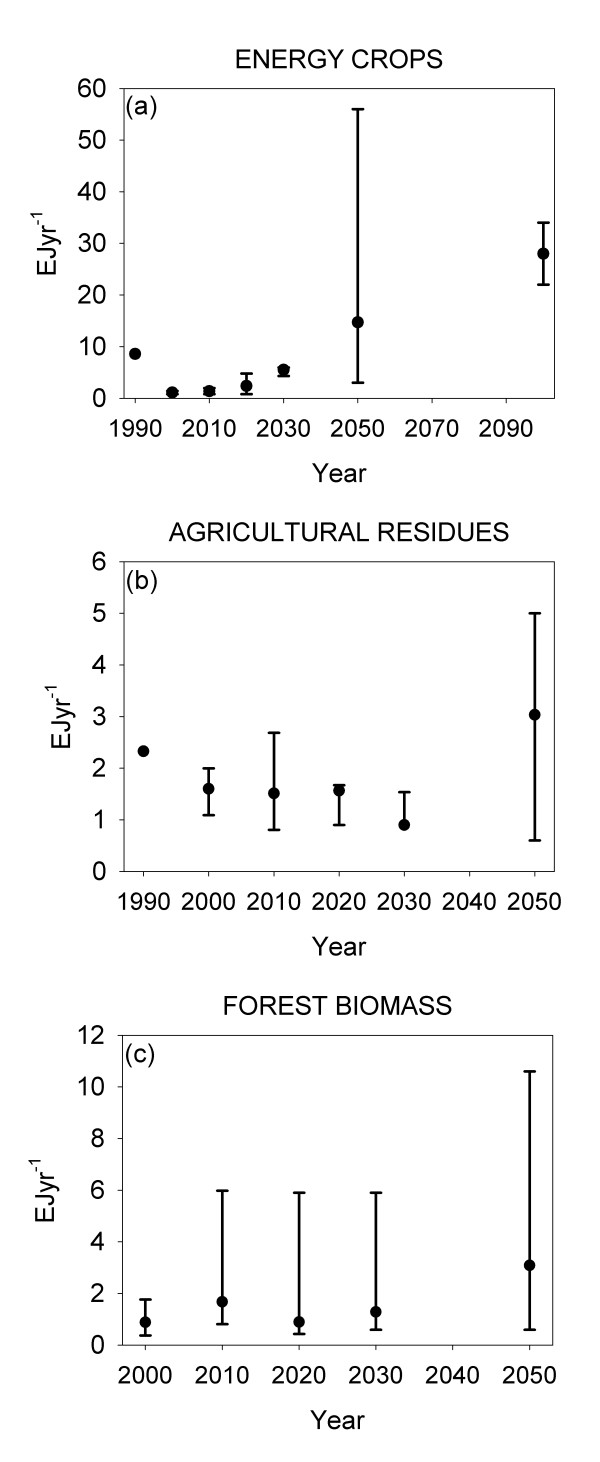
**Median and range of the potential from three major sources of biomass for energy.** Data on energy crops are based on [16,18,23,30,41,50,53,54], agricultural residues based on [16,18,30,39,41,44,49,50,53,55,56], and forest biomass based on [18,31,35,39,41,50,53,54,56,59,60].

**Figure 3:**
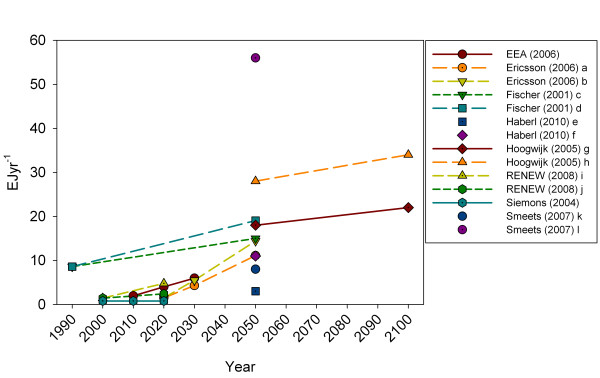
**EU25/27 Energy crop potential from 1990 to 2100 from individual studies.** Letters a-f signifies different scenarios. a - low biomass harvest; b - high biomass harvest; c - min; d - max; e - Scenario S1, maximised biofuel production by 2020; f - Scenario S2, self-sufficient biofuel production by 2020.

Dedicated energy crops are expected to make up a major part of future bioenergy supplies (Figure 2a). Estimates of the current (~2010) resource range from 0.8–2.0 EJyr^−1^, increasing to 4.3–6.0 EJyr^−1^ in 2030, 3–56 EJyr^−1^ in 2050 and 22–34 EJyr^−1^ by the end of this century. The overall increasing trend is supported by individual resource estimates over time (Figure 3).

Residues from agricultural production e.g. cereal straw, corn stover and rape straw is a readily available resource from already managed land. Estimates of the current resource range from 0.8 to 3.9 EJyr^−1^ (Figure 2b). There is no unambiguous trend in estimates towards increasing or decreasing resources in the future. 2030 estimates range from 0.9 to 3.1 EJyr^−1^ and 2050 estimates range from 0.6 to 5.0 EJyr^−1^.

Forest biomass constitutes felling residues and wood biomass from early thinning and stand management. The current forest bioenergy potential shows the largest variation, with estimates between 0.8 and 6.0 EJyr^−1^ (Figure 2c). 2050 estimates range from 0.8 to 10.6 EJyr^−1^. The EU27 countries also have potential biomass resources from already processed biomass. These resources are highly diverse and constitute e.g. sewage sludge, municipal solid waste, wood processing residues, manure, other agricultural wastes, and food processing waste. The European Environmental Agency (EEA) assesses the secondary biomass resources to 3.1 EJyr^−1^ in 2010, growing to 3.2 EJyr^−1^ in 2030. Focussing on wood industry residues Ericsson et al. [41] assess the resource for the EU25 to 1.1 EJyr^−1^ from 2020 past 2040, Mantau et al. [60] find an EU27 potential of 1.0 EJyr^−1^ in 2010 and 1.3 EJyr^−1^in 2030. In comparison the EEA [42] assessment of wood processing residues find an EU25 potential of only ~0.4 EJyr^−1^.

## Discussion

### Energy crops

**Figure 4:**
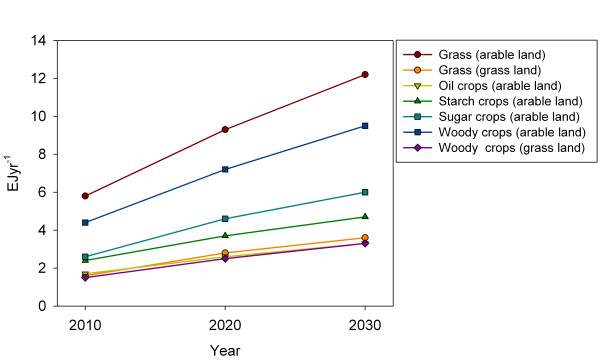
**Development in the potential of different dedicated energy crops from 2010 to 2030 on arable land or grass lands respectively.** Data from [54].

Currently energy crops in Europe comprise mainly traditional food crops as rape seed and sugar or starch crops [61]. In future supply scenarios lignocellulosic energy crops are expected to play a larger role. de Witt et al. [54] finds that the potential from traditional food crops will increase with 7.3 EJyr^−1^ from 2010 to 2030, whereas the potential from lignocellulosic crops will increase with 15.3 EJyr^−1^ (Figure 4).

### Agricultural residues

Agriculture in the EU27 countries is characterised by a high proportion of cereal production. In 2007 56% of arable lands were grown with cereals [62]. Thus agricultural residues comprise mainly straw, leaves and stalks from grass species (*Poaceae* family) as e.g. wheat, maize, barley and rye.

### Forest biomass

The EU27 countries have experienced a steady increase in wooded land of more than 22 Million ha over the past more than 20 years [63] and resources from forestry and forest industries are a main contribution to bioenergy production. At present forest biomass is mainly used to support material demand, however, between 2010 and 2020 energy purposes may take over as the major demand [60]. The potential supply of biomass from forests, stems, felling residues and bark is not expected to change significantly from 2010 to 2030, but the potential from wood industry residues will increase some 30% in the same period [60].

### Development trends

Many of the reviewed studies point to short rotation (1–10 years) energy crops as the main future resource of biomass for energy purposes. A reason for this probably relates to the fact that such crops fit in the current EU Common Agricultural Policy’s financial support schemes. Short rotation crops, however, are only some among many options for a future bioenergy supply. Tree species in longer rotation may also show interesting perspectives as energy crops. Crops in short or long rotation exhibit different characteristics in terms of flexibility regarding crop renewal, flexibility at harvest, storability, productivity and growth pattern. Combining the characteristics of different crops may prove beneficial in the development of a secure and productive bioenergy supply.

## Variability and inconsistencies in biomass potential studies

Studies on biomass/bioenergy resources apply different methodologies, different definitions of resource potential, different geographical scope and different assumptions regarding availability. The lack of methodological consensus leads to disagreement, i.e. different potentials among different studies as to how much biomass of various fractions is available for energy. Resource potentials are usually categorised as theoretical, technical, economical or sustainable with potential sub-grouping relating to the practical implementation of a given potential within a certain time frame. Theory suggests a ranking of potentials as:

Theoretical > technical > economic > sustainable.

Comparing results from different assessments of the same biomass resource doesn’t provide a clear picture of the above relation. Variability and methodological inconsistency seem to overrule the theory.

Data illustrated here (Figures 2 and 3) represent the technical potential of biomass for energy. ‘Technical potential’ can be defined as *“the fraction of the theoretical potential, which is available under the regarded techno-structural framework conditions and with the current technological possibilities. Spatial confinements due to competitions with other land uses as well as ecological and other non-technical constraints are also taken into account”*[64]. The term ‘technical potential’ is not used unambiguously in literature [28,58] but the above definition covers most applications.

2Böttcher et al. [39] report theoretical and technical potentials of agricultural residues and find the theoretical potential to 2.7 EJyr^−1^ and the technical potential to 0.8 EJyr^−1^. Böttcher et al. [39] also show for forestry a theoretical potential of wood residues of 5.2 EJyr^−1^ and a technical potential of 3.3 EJyr^−1^. Correspondingly, Asikainen et al. [59] find a theoretical potential of forest energy to 7.1 EJyr^−1^ and technical 1.7 EJyr^−1^. Mantau et al. [60] show technical to theoretical potential relations of forest biomass in 2010 of 6.5:11.1 and in 2030 of 6.4:10.9. A significant component in the difference between theoretical and technical potential is the omission of stump harvesting. While there is agreement that the technical potential is much lower than the theoretical potential, disagreement prevail on the fraction by which technical potential make up the theoretical potential. Here we show fractions between 24% and 63%.

Some references report sustainable potentials of forest biomass. Hetch [46] show for the EU27 a current potential of 1.4 EJyr^−1^, Fischer et al. [16] find for geographical Europe excluding former Soviet Union the current potential to 11.3 EJyr^−1^ rising to 14.2–18.1 EJyr^−1^ in 2050. EEA [42] finds for the EU25 a current potential of 1.8 EJyr^−1^ decreasing to 1.6 EJyr^−1^ in 2030. Although the geographical coverage isn’t identical, the above data illustrate the level of variation between individual assessments even though the scope (sustainable bioenergy potentials from forest biomass) is the same.

### Geographical scope

Geographical scope differs among studies and impedes direct comparison among them. Data presented in Figures 2,3 and 4 represents EU25, EU27 or EU27 and Switzerland. Johansson et al. [47] estimate for OECD Europe agricultural residue potential of 1.41 EJyr^−1^ in 2025 and 2050 and a forest + forest industry potential of 1.69 EJyr^−1^ in 2025 and 1.68 EJyr^−1^ in 2050. Also for OECD Europe Bauen et al. [38] find a crop residue potential of 3.4 EJyr^−1^ and a forest residue potential of 4.8 EJyr^−1^ in 2020. Hall et al. [45] also look at OECD Europe and find a 2020 residue potential of 2.3 EJyr^−1^ and energy crops of 6.7 EJyr^−1^. Estimates for OECD Europe do not deviate unequivocally from EU27 estimates. Bauen et al’s estimates on agricultural and forest residues exceed the ranges plotted in Figure 2. Energy crop potentials as estimated by Hall et al. exceed the plotted ranges. de Witt et al. [54] estimate the biomass for energy potential for various resources for the EU27 and Norway, Switzerland and Ukraine. Here dedicated energy crops and agricultural residues exceed the plotted ranges, whereas forest biomass falls within the range. The inclusion of a country with a large agricultural sector may be one of the reasons for the comparatively high estimates of resources from agricultural lands. Rettenmaier et al. [64] have made an attempt to calibrate a range of recent bioenergy resource assessments regarding Europe to EU27 level to improve comparability across individual assessments.

### Sustainability constraints

It is widely acknowledged that ecological concern and competing uses constrain the use of the agricultural residue resource and all studies apply an availability factor to the total residue production. There is, however, no general agreement among studies on the size of these constraints and thus the factors used. Literature reviewed here apply availability factors of 25% [30,41], 30% [39,50,56], 40–50% [49] and 50% [44,54]. A number of studies do not specify what availability factor is used [16,18,42,53]. There is no significant correlation between the availability factor used and the residue potential reported (data not shown). Other literature presents availability factors ranging from zero [65] to 40–50% [66] to 60% [67]. Ecological availability of agricultural residues cannot be determined with general validity [68]. Positive correlations between carbon content in soil and soil productivity have been found for various crops in different regions [69-72]. It is shown that differences in crop yield induced by differences in soil organic carbon content in many cases can be overcome by appropriate supply of mineral fertilisers [73,74].

Matau et al. [60] quantify the impact of environmental constraints on the potential of various biomass fractions from forestry. The reduction in potential of logging residues due to environmental constraints vary across regions and fall for most parts of Europe between 33 and 50%. However in some regions e.g. northern Scotland, the alpine region and northern Scandinavia it may reach 100%. Nabuurs et al. [75] find that meeting European renewable energy targets may increase the harvesting pressure on European forests and lead to overharvesting in some regions specially around 2030–2040.

### Methodological challenges

The scientific challenge, as we see it, is not that different biomass resource assessments return differing results. Resource assessments are used to answer different questions, e.g. on the impact on energy production in general of enacting certain policies; on the availability of specific resources for specific purposes; or on the viability of shifting from one energy resource to another. Consequently, the answers differ. The challenge is rather the lack of reproducibility and transparency. Resource assessments aiming at the same question should preferably return comparable answers. In the framework of the Biomass Energy Europe project (http://www.eu-bee.org) European bioenergy potential studies have been analysed to develop a harmonised resource assessment methodology [64].

## Biomass demand

**Figure 5:**
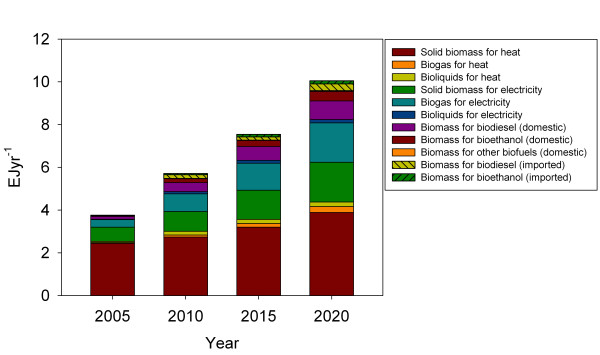
**Estimated demand for biomass for energy in the EU27 countries based on national renewable energy projections[76] and reported conversion efficiencies [5,77].**

National renewable energy action plans provide an estimate of domestic and imported biomass resources required to meet the targets of the EU energy strategy. These estimates are reported under different assumptions regarding conversion efficiency and in differing units hampering direct use of data. We calculate biomass demand on basis of national renewable energy projections [76] with the application of conversion efficiency multipliers based on [5,77] and the assumption that 11% of European electricity generation is based on co-generation of heat and electricity [78]. We find that the amount of biomass required meeting the EU27 targets increase from 3.8 EJ in 2005 to 10.0 EJ by 2020 (Figure 5). In 2009 the EU27 countries had a primary production of biomass and waste of 4,2 EJ [79]. The amount of biomass required in 2020 includes the use of ~0.1 EJ of traditional food crops, i.e. cereals and sugar beet for 1^st^ generation bioethanol and oil crops for biodiesel as well as 0.5 EJ in countries outside EU27 as feedstock for liquid fuel. Heat and electricity production make up the lion’s share in all years to 2020, but transportation increase from 5% of the biomass demand in 2005 to 18% in 2020. In a longer perspective to 2050 or 2100 substantially more biomass must be expected to be converted to energy services in the EU27 countries to meet the long term targets of decarbonising the electricity and transport sector [2].

Demand for biomass for other purposes besides energy has an impact on the bioenergy potential. Exploring four different development scenarios Hoogwijk et al. [23] find that the meat demand has an impact on area available for energy crops. In their high population growth and high meat consumption scenario (A2) they show close to three times the agricultural area for food production than their low population growth and low meat consumption scenario (B1) reducing the European energy crop potential on abandoned agricultural land from 24 to 21 EJyr^−1^ in year 2100. On a global scale Yamamoto et al. [80] finds that high demand for meat reduces the global energy crop potential from 150 EJyr^−1^ (reference case) to 78 EJyr^−1^ (high demand for animal food case), whereas the potential from food biomass residues increase from 160 to 186 EJyr^−1^ for the same scenarios.

## Land use

Bioenergy production may be increased through further mobilisation of existing resources, intensification of current production or expansion into ‘new’ land. The impact on GHG emissions from an expanded production of energy crops have been widely analysed, e.g. [81,82]. Delucchi [83]reviews recent literature and methodologies. One main finding is that the conversion of forest, grasslands and wetlands to agriculture results in loss of carbon in soil and biomass. This is corroborated by Don et al. [84]. The type of biomass for energy grown on converted land also affects the carbon balance in soil and biomass. In general perennial species as sugar cane and *Miscanthus* are favourable to annual species as they sequester carbon in soil [85]. An option for reducing the impacts of bioenergy on land use may be to consider a more integrated approach to land use simultaneously producing energy and food on the same land [86,87].

Burney et al. [88] and Vlek et al. [89] find that in many cases increased agricultural production achieved through intensification is favourable to agricultural expansion in terms of GHG emissions. This conclusion, however, requires land liberated due to intensification are converted into forest of forest land being ‘spared’ from conversion.

Land use, land use change and (environmental) sustainability of energy crop production have become a major issue in European policy [90] on biomass for energy. As a result GHG emissions caused by land use change must now be included in meeting sustainability criteria set up by the European Parliament [1]. Land use change is highly dynamic and localised and the quantification of land use effects on the climate currently builds on a weak methodological foundation [91]. Models tend to be static and work on a national or regional geographical and not validated against local empirical data [84,91].

Ovando et al. [92] estimate the land required to produce energy crops in EU-25 based on a number of studies. 5–20 Mha is required in 2000–2010 rising to 25–45 Mha in 2100. The European Biomass Association estimates the current (2007–08) allocation of traditional food crops to energy purposes to 4 Mha and additional 85,000 ha to lignocellulosic crops [93]. While area availability is the ultimately limiting factor for expanded production of energy crops, limited access to water and nutrients may also constrain bioenergy potentials. Particularly nitrogen efficient crops are found among tree species and grasses [94,95].

### Component specificity

**Figure 6:**
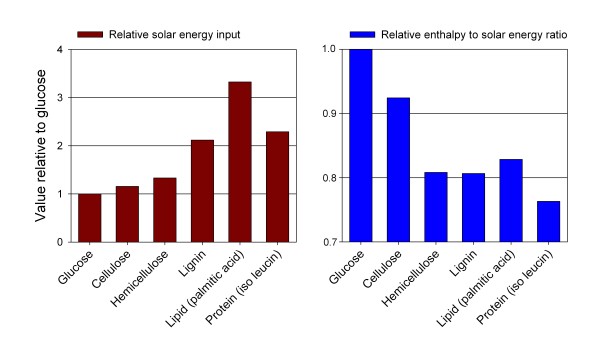
**Solar radiation energy needed to the synthesis of individual plant components (left pane) and the relation between solar energy input and enthalpy of combustion of individual plant components (right pane).** Values are relative to energy input and enthalpy to energy input ratio for glucose. Based on [96-98].

Using biomass for energy is a way of harnessing solar energy. Biomass is a complex mixture composed primarily of more or less polymerised sugars, lipids, lignin, proteins and organic acids in varying proportions [96]. Complex components require more solar energy for synthesis than simple molecules and structures. Synthesis of lignin, lipids and proteins theoretically requires respectively 112, 233 and 129% more solar energy input per unit of mass than the synthesis of starch (calculated from [97,98]) (Figure 6 left pane). These components also contain more chemically bound energy but the relation between energy content (enthalpy of combustion calculated from [99,100]) and required solar energy input show that, relatively speaking, the use of proteins for energy generation is a poorer exploitation of solar energy and thus land area, than the use of lignin, lipids and hemicellulose, which again is a poorer exploitation of solar energy than the use of cellulose (Figure 6 right pane).

Some conversion technologies have a degree of component specificity, while others do not. Thermochemical conversion destroys every component in biomass converting it to CO_2_, CO, H_2_, CH_4_, NO_X_ and water in various amounts [101]. Methane fermentation technologies exhibit higher specificity. Lignin is not converted, 34–92% of the proteins are hydrolysed and fermented depending on various conditions [102], 70–95% of lipids [103,104], 65–70% of polymerised sugars [103] and ~95% of sugar oligomers are destroyed [103]. In ethanol fermentation only simple sugars are converted to ethanol and CO_2_. Conversion rates of polymerised sugars depend on the efficiency of the preceding hydrolysis. Lignin and protein are not destroyed during fermentation.

The higher component specificity of biochemical and catalytic-chemical conversion has the attraction over thermochemical conversion of conserving to some degree the components requiring more solar energy for biosynthesis. Preserving the solar energy intensive components to uses other than energy may reduce the impact on land use from bioenergy production. Proteins found in the by-product from grain based ethanol production may serve as valuable feed product [105] but may also be further processed to higher value products and provide an even higher displacement of fossil fuels [106]. In a biorefinery context the lignin residue from processing lignocellulosic biomass is considered a valuable energy source for process heat and electricity. Industrial processing of lignin to aromatics as phenol, styrene or toluene may, however, also provide higher abatement of fossil GHG emissions [106].

## Conclusions and outlook

The demand for biomass for energy in the European Union will increase from the current 5.7 EJ yr^−1^ to 10.0 EJyr^−1^ by 2020. Dedicated energy crops grown on liberated agricultural land or marginal lands are expected to be able to meet the major part of the increasing biomass demand. Residues from agriculture and forestry are not expected to increase significantly in the future. The demand for biomass for energy will probably increase also beyond 2020 and not only in Europe. This calls for further technology development and increased focus on technology integration to meet the grand challenge of a decarbonised energy supply.

Further development of harmonised and transparent assessment methodologies is required to improve applicability and reproducibility of such assessments.

To meet future needs for biomass, not only for energy, also for food, feed and materials emphasis must be put on increasing the production of biomass per unit of land and exploring the potentials in new biomass sources to reduce the pressure on native and protected eco systems.

Further focus on optimised utilisation of individual plant components in biomass with a link to the component specificity of different conversion technologies could improve the utility gained from biomass and bioenergy and reduce negative impacts on land use.

## Endnotes

^a^The current (2012) 27 member countries of the European Union: Austria, Belgium, Bulgaria, Cyprus, Czech Rep., Denmark, Estonia, Finland, France, Germany, Greece, Hungary, Ireland, Italy, Latvia, Lithuania, Luxembourg, Malta, Netherlands, Poland, Portugal, Romania, Slovakia, Slovenia, Spain, Sweden, United Kingdom.

^b^EU25 = Members of the European Union as of 2004: EU27 minus Bulgaria and Romania.

## Competing interests

The authors declare no competing interests.

## Author’s contributions

NSB and CF conceptualised the manuscript. NSB was responsible for literature review, data acquisition and analysis and initial writing. CF contributed with review and editing. NSB and CF read and approved the final manuscript.
